# Dream to Predict? REM Dreaming as Prospective Coding

**DOI:** 10.3389/fpsyg.2015.01961

**Published:** 2016-01-05

**Authors:** Sue Llewellyn

**Affiliations:** Faculty of Humanities, University of ManchesterManchester, UK

**Keywords:** prediction, prospective coding, REM dreaming, pattern, unconscious

## Abstract

The dream as prediction seems inherently improbable. The bizarre occurrences in dreams never characterize everyday life. Dreams do not come true! But assuming that bizarreness negates expectations may rest on a misunderstanding of how the predictive brain works. In evolutionary terms, the ability to rapidly predict what sensory input implies—through expectations derived from discerning patterns in associated past experiences—would have enhanced fitness and survival. For example, food and water are essential for survival, associating past experiences (to identify location patterns) predicts where they can be found. Similarly, prediction may enable predator identification from what would have been only a fleeting and ambiguous stimulus—without prior expectations. To confront the many challenges associated with natural settings, visual perception is vital for humans (and most mammals) and often responses must be rapid. Predictive coding during wake may, therefore, be based on unconscious imagery so that visual perception is maintained and appropriate motor actions triggered quickly. Speed may also dictate the form of the imagery. Bizarreness, during REM dreaming, may result from a prospective code fusing phenomena with the same meaning—within a particular context. For example, if the context is possible predation, from the perspective of the prey two different predators can both mean the same (i.e., immediate danger) and require the same response (e.g., flight). Prospective coding may also prune redundancy from memories, to focus the image on the contextually-relevant elements only, thus, rendering the non-relevant phenomena indeterminate—another aspect of bizarreness. In sum, this paper offers an evolutionary take on REM dreaming as a form of prospective coding which identifies a probabilistic pattern in past events. This pattern is portrayed in an unconscious, associative, sensorimotor image which may support cognition in wake through being mobilized as a predictive code. A particular dream illustrates.

## Introduction

There is a long standing idea that, based on prior experience, the brain generates predictions (or expectations) to interpret and, thereby, identify sensory input (Helmholtz, 1909; Gregory, 1980). Equally, the notion that expectations link perception and action is an old one (James, 1890). These revived ideas have excited current interest in the predictive brain across multiple areas (Bubic et al., 2010). Specifically, in the brain as a “Bayesian inference machine” (Knill and Pouget, 2004; Yuille and Kersten, 2006; Friston, 2010), in bi-directional “hierarchical generative models” (Dayan et al., 1995; Rao and Ballard, 1999; Friston, 2012; Clark, 2013), and in predictive (Friston, 2005; Friston and Kiebel, 2009) and prospective coding (Rainer et al., 1999; Ferbinteanu and Shapiro, 2003; Schütz-Bosbach and Prinz, 2007). Against this background, Yordanova et al. (2012) call for a major research focus on the predictive brain during altered states of consciousness, suggesting REM sleep may be of particular consequence.

This paper focuses on the *form* coding may take, hypothesizing that, a REM dream constitutes a form of prospective image-based code which identifies an associative pattern in past events and, therefore, portrays associations *between* past experiences (rather than the experiences as such). This image-based code may be retained at an unconscious level and mobilized to predict the immediate sensory environment and interpret the causes of sensory input during wake.

In relation to this hypothesis, two points are worthy of note. First, there is agreement with Stickgold (2002) who argues that, in the absence of sensory input, reactivated memories must be the source material for dreams. Albeit this paper hypothesizes that REM dreams portray only those elements of reactivated memories which identify an associative pattern in past events. Second, Horne (2013) presents evidence that REM sleep prepares for wake, here this proposal is extended to REM dreams also- in the sense that REM dreams are argued to create prospective codes which impact on perception and action in subsequent waking states.

### Patterns, predictions, and unconscious inference

In evolutionary terms, any animal which can move must predict where food, predators, and mates can be found (Schultz et al., 1997). Prediction can preclude possible futures and, thus, would have enhanced fitness and survival (Gilbert and Wilson, 2011). For example, if memory of a predator sighting interprets sensory input as indicative of a predator, the animal can take avoiding action and preclude being killed on this occasion. This perspective on memory as inherent to sense-making extends to an enactivist ontology, where the world, as experienced, is sculpted by expectations which project meaning and propel willed actions (Varela et al., 1991; Di Paolo, 2009).

Predictions, on the basis of memories of past events, are only possible if events are patterned in some way (Panichello et al., 2012). Memory is fundamentally associative in both structure and process (Fuster, 1997, 1999). Associative structure and process may have evolved to embed patterns in memory. In turn, associative activation of memories generates the patterns which form predictions (Bar, 2007, 2011; Aminoff et al., 2008; Buckner, 2010). Patterns may be deterministic, generated by fixed regularities, co-occurrences or associations. For example, night with associated dark follows and is associated with day, with its associated light. On the other hand, the patterns generated by the behavior of living beings will be probabilistic, based on regularities or associations which have a tendency to co-occur (see later discussion). Probabilistic patterns may be difficult to discern because they are based on memories of infrequent, complex or non-obvious co-occurrences or associations.

Animals are thought able to discern patterns in events because they are capable of inference (Tolman, 1948; Pfeiffer and Foster, 2013), through using configural, as opposed to elemental, associations (Honey et al., 2014) and can link common elements across different experiences (Wood et al., 1999; Hampson et al., 2004) to discern patterns in events. Humans too associate common experiential elements in a combined representation (Zeithamova and Preston, 2010), such integrated representations portray memory elements in “prospectively useful formats” (Zeithamova et al., 2012). Animals lack sophisticated language skills, however. Both animals and early humans would have “thought” non-verbally through images (Baumeister and Masicampo, 2010). The primary function of mental imagery may be to generate predications (Moulton and Kosslyn, 2009). An image-based code may portray the associations which reflect experiential patterns. These associations drive expectations for sensory input during wake. Clearly, at any point in time in wake, the sensorium is infinite and, in consequence, must be sampled. Image-based predictions may drive “active sampling of sensory data” to identify its expected causes (Friston et al., 2012). Expectations about causes can only be on the basis of inferring a causal pattern in the world.

Helmholtz (1909) proposed that unconscious inference is the foundation for perception. For humans and most other mammals, maintaining visual perception is crucial. Unconscious image-based predictions would not interfere with visual perception. They would also facilitate rapid action and be particularly relevant in situations of threat and potential danger. Dangers require fast processing and rapid action (Öhman et al., 2000; Carretié et al., 2005). In evolutionary terms, early humans had to visit particular places (associated with survival). Such landmark places held rewards but also dangers. For example, a waterhole where “sit and wait” predators, like lions, may seek to ambush prey in the surrounding vegetative cover (Hopcraft et al., 2005). On approach to a waterhole, unconscious image-based predictions, based on prior predator sightings, may drive active sampling of color and movement in the vegetative cover- if a predator was judged to be a plausible explanation for sensory input. Visual perception has been found to be predictive and probabilistic rather than passive, incoming visual input is interpreted and fine-tuned unconsciously with reference to memories, including memories of prior beliefs (Kersten et al., 2004; Knill and Pouget, 2004).

Congruent with the idea that expectations link perception and action, an unconscious image-based code may also portray memories of action appropriate to the circumstances. The activation of mnemonic images enables unconscious bodily responses which reflect the situation i.e., mnemonic images are fundamentally efferent processes, the image is essential for the motor actions, for review, see Cuthbert et al. (1991). Indeed, motor directives can be integrated into sensory perceptions to facilitate rapid action, for review see Bubic et al. (2010). Predictive coding enables the motor system to “select appropriate responses” before an anticipated event is realized (Schütz-Bosbach and Prinz, 2007).

### Prospective coding and predictive coding

Bubic et al. (2010) comment that the terms “predictive” and “prospective” are not consistently used in the same way but, on the other hand, are not always clearly differentiated, a situation that can lead to confusion, they suggest that “predictive coding” is taken to mean that, during the wake state the brain anticipates upcoming sensory inputs and actively samples external stimuli to identify their causes, rather than registering stimuli in a passive manner. In this article predictive coding in wake is hypothesized to rely upon unconscious, internally generated prospective codes. Prospective coding is understood here as the off-line creation of prospective codes which are oriented toward the future and can be mobilized as predictive codes to interpret the immediate environment during wake.

A key aspect of prospective codes is that because they portray associative visual patterns *between* prior events (rather than the events *per se*), therefore, they engender representations that are quintessentially fictive or counterfactual. In this sense, this paper equates prospective coding with the encoding of counterfactual representations which can inform in the future, which is one step further than classical predictive coding theory (that only furnishes predictions of the current outcome given their hypothetical causes).

REM dreaming is argued to be conducive to prospective coding. Such coding may require the core characteristics which REM dreaming shares with the wake state: active consciousness during which a world appears with (almost always) an embodied, agential self at its center (Llinás, 2002; Metzinger, 2009).

## REM sleep and dreaming as prospective coding

During off-line states, people contemplate the past to anticipate the future (Bar, 2007, 2011; Schütz-Bosbach and Prinz, 2007). Sleep is clearly off-line. As a whole, sleep preferentially reactivates memories associated with future rewards (Fischer and Born, 2009) and memories expected to be relevant to future behavior (Wilhelm et al., 2011). Hobson et al. (2014) argue the virtual realities engendered in REM dreams refine predictions. A REM dream image may be the result of prospective coding which refines predictions through hyperassociating elements of different past events during the identification of a personally significant pattern which arises from these elements of past events.

Dreaming hyperassociates different memories (Hobson, 2002; Llewellyn, 2013; Horton and Malinowski, 2015; Malinowski and Horton, 2015). Whole memories are hardly ever replayed in dreams (Fosse et al., 2003; Malinowski and Horton, 2014), rather elements of experiential memories are associated in visual scenes (Hobson, 1988; Hartmann, 1996; Walker and Stickgold, 2010). Memory trace reactivation in REM was associated with the extraction of patterns in higher order information (Peigneux et al., 2003). Using fMRI, Chow et al. (2013) found brain connectivity during REM to be consistent with the extraction of patterns from past events. REM sleep selectively processes personally-significant material (van Rijn et al., 2015). The extraction of any personally meaningful pattern in past experiences would enable expectations about future, personally significant, events.

Personally significant, past and future events will be emotionally charged. Indeed, anticipatory affect may be an integral aspect of prediction (Barrett and Bar, 2009; Anderson et al., 2011). Emotional memory is enhanced in REM sleep (Cahill and McGaugh, 1998; Wagner et al., 2001; Nishida et al., 2009) and heightened emotional tone strengthens memory associations (Cahill et al., 1996; Hamann et al., 1999). REM sleep may preferentially reactivate emotionally significant memories (Sterpenich et al., 2007, 2009). About 75–95% of dreams depict emotional events, such dreams are largely during REM sleep (Hobson et al., 1998, 2000). REM dreams are characterized by fear and anxiety (Smith et al., 2004). Sleep deprivation (whole night) leads to preferential retention of negative (rather than positive) memories (Walker and Stickgold, 2006; Sterpenich et al., 2007; Yoo et al., 2007; Walker, 2008) and REM sleep even counteracts the suppression of unwanted memories (Fischer et al., 2011). The preferential retention of negative, rather than positive memories, after sleep deprivation, may have an evolutionary explanation. Animals face many survival challenges in natural environments, early humans, too, would have experienced such testing conditions. An ability to predict threats to survival would have been adaptive, therefore, evolution may have prioritized negative over positive memories—*under conditions of sleep deprivation*.

Evolutionary explanations for REM sleep functions have been suggested. Evolutionarily ancient brain networks are preferentially engaged in REM sleep, for review see (Doricchi et al., 2007). In humans and most mammals, brain processes in REM indicate behaviors such as exploration, foraging, eating, and spatial navigation and the ability to deal with fear and anxiety, suggesting that REM serves ecological purposes and had an evolutionary imperative (Horne, 2013, 2015).

As noted earlier, in evolutionary terms, the rewards of food or water were often only obtained under situations of risk because places, such as waterholes and dependable food sites, where many species congregated, both provoked competitive interactions and also attracted predators. In such circumstances, simple predator and competitor avoidance was not possible. To obtain food and water the animal would have been exposed to risk. Predicting the patterns of behavior of both competitors and predators would have reduced risk and increased the chances of securing the rewards.

Rewards, dangers and competitive interactions were emotive and of high personal significance. Wallas (1926) identified an evolutionary imperative to recognize “resemblance in difference” i.e., “hidden” or non-obvious likenesses between, for example, different kinds of scraps which are all associated because they are all food and animals from different species which are all associated because they are all competitors or predators. Likenesses, whether obvious or non-obvious, constitute patterns in events, so do place-object associations. With regard to predators, it is not only necessary to predict “Is this a predator”—to identify it from what may be ambiguous sensory input but also to predict “How does the predator behave” and “Where and when can it be found”—to take appropriate action to avoid it.

Predator sightings (the object) in, for example, three out of ten approaches to a waterhole (the place) would have been a personally significant pattern which could give rise to a probabilistic prediction. Even if the pattern of sightings of a predator was regular, for example on the third, sixth and ninth visits, predictions about the behavior of this predator, would still be probabilistic because patterned behavior can always vary. A pattern of predator sightings on three out of ten approaches to a waterhole may be driven by another non-obvious, meaningful association. For example, the predator only visits this waterhole when it anticipates that prey will be abundant. In such a circumstance, identifying an association between “waterhole” and “abundance of prey” and “presence of a predator” would enhance survival because the animal could visit at less populous times. As mentioned earlier, unconscious predictions are particularly relevant in situations of potential danger.

However, unconscious predictions are also important whenever speed is crucial for success. For example, identifying a probabilistic pattern in the presence of mates at a landmark place would enhance reproduction and speed would be necessary to secure a mate against competitors. Unconscious learning of probabilistic patterns to predict future events benefits from sleep (Djonlagic et al., 2009) and is REM sleep dependent (Barsky et al., 2015).

Several studies indicate that REM sleep enables the identification of meaningful patterns. Walker et al. (2002) found a 32% increase in the number of anagrams solved after REM awakenings, as compared to non-REM (NREM). There is also evidence that the patterns identified in REM may be “loose” or non-obvious. For example, when awakened from REM sleep, subjects demonstrated more priming in response to looser, non-obvious primes (e.g., thief-wrong) as compared to stronger, obvious primes (e.g., hot-cold), whereas after NREM awakenings subjects showed more responsiveness to the stronger, obvious primes (Stickgold et al., 1999). REM, as compared to quiet rest in wake and NREM sleep, increased subjects' abilities to think of a word linked with three test words that seemed unrelated, for example, “surprise,” “line,” and “birthday” do not seem associated but “party” is connected with all three (Cai et al., 2009). Looking for non-obvious patterns in past events involves making creative (i.e., non-obvious or loose) associations between experiences. Sterpenich et al. (2014) found that reactivating memories during REM sleep engendered new creative associations amongst them.

Cognitive control and tight, analytic reasoning depend upon the prefrontal cortex (Nathaniel-James and Frith, 2002; Goel and Dolan, 2004; Ridderinkhof et al., 2004; Reverberi et al., 2005a), which is deactivated during REM sleep (Maquet et al., 1996; Braun et al., 1997; Maquet, 2000). For a non-obvious insight task, 82% of patients with focal damage to the frontal lateral cortex solved the task whereas only 43% of healthy participants did so (Reverberi et al., 2005b), indicating that prefrontal areas actually impede non-obvious associative insights—possibly through exercising cognitive control which biases toward more obvious associations. Similarly, Limb and Braun (2008) argued that de-activation of the frontal cortex may generate “spontaneous unplanned associations,” see also (Liu et al., 2012).

Association between memories is driven by inferring their meaning. Experiences (or things) are associated in the mind/brain when their meaning is the same or similar. For humans, meanings may be culturally shared (Bruner, 1990) but meaning is often personally specific i.e., what does this mean *to me*. Carruthers and Ziolkowski (2002) point out that associational meaning, is often individualized rather than universal, dependent on one's prior experiences, and personal needs, desires and goals. For example, “hospital” may be most meaningfully associated to “eye” to me but “recovery” to another or even “career” to some. Meaning can also imply concrete significance for needs, desires or goals (Clore and Ortony, 2000) i.e., what does this mean *for me*?

Such personal meanings may have evolved from the relational “affordances” which link animals and their environment (Chemero, 2003). From a relational view, environmental affordances only become resources if there are animals to take advantage of them, for example, fruit only means food *for* fruit eating animals, see Turvey (1992). To exploit what the environment affords *for me* it is sometimes necessary to avoid threats, archetypically, from competitors and predators.

In a natural environment, a lion means “fundamental threat for my goal of survival.” The presence of a competitor means “rivalry for resources.” Memories of possible predator sightings on approach to waterholes would have been associated because they all meant and implied the same motor actions of rapid flight or freezing. Equally, memories of competitors at dependable food sites all meant potential struggle. Or memories of mating at a particular place all meant satisfaction of instinctive drives. In their entirety, memories of visits may have been completely different but with respect to the element “predator sighting,” “competitor present” or “mating opportunity,” respectively, they were the same. In consequence, a prospective code, created in REM dreaming, which identifies a pattern in “predator sightings,” “competitors present,” or “mating opportunities” may discard much that is already predictable about the visits to associate only memory elements relevant to “predator sightings,” “competitors present,” or “mating opportunities.”

As mentioned above, if a conscious image-based predictive code was mobilized in wake it would interfere with visual perception, and be detrimental in situations where rapid action is essential. If any insights into patterns are acquired unconsciously in wake before sleep, REM sleep stabilizes them at an unconscious level (Yordanova et al., 2008). Implicit learning seems to be especially sensitive to REM sleep deprivation (Smith, 1995; Plihal and Born, 1997) and REM sleep benefits implicit visual memories (Wagner et al., 2003).

Indeed, throughout evolutionary time, “vision for action” may have relied on unconscious mental imagery (Goodale and Milner, 1992, 2013; Milner and Goodale, 2008). Congruent with the idea that the predictive brain links expectations with perception and action (see above), the imagery during REM dreaming is sensorimotor in nature and action-oriented (Hobson, 2002; Llinás, 2002). As discussed above, REM dreaming is emotionally charged, particularly with the primary emotions of elation, fear and anxiety (Hobson, 1988, 2002). Such emotions may have evolved from securing rewards in situations of risk. From an evolutionary perspective, emotions are action depositions and reflect two fundamental opposed action sets: approach, following positive evaluations and avoidance, after negative evaluations (Lang et al., 1990; Lang, 1995; Elliot and Church, 1997; Carver et al., 2000; Elliot, 2006). These evaluations of positive or negative consequences can be unconscious (Chen and Bargh, 1999; Neumann et al., 2003; Bargh and Morsella, 2008).

Memory reactivations in post-training REM sleep optimize visuo-motor responses (Laureys et al., 2001), suggesting that REM dreaming may rehearse the motor actions necessary for the approach or avoidance behaviors appropriate to the identified pattern in events [c.f. the “dream as rehearsal” of motor programs for instinctive, particularly threatening experiences in wake (Jouvet, 1999; Revonsuo, 2000)]. Such processing may enable the integration between expectation, perception and action which is thought to characterize predictive coding in wake.

### Visual processing, REMs and prospective coding

Certain visual processing abilities e.g., identifying embedded figures and recognizing images in unfocussed pictures are the best predictors of solving problems requiring insight into patterns (Schooler and Melcher, 1995; Bowden et al., 2005). In evolutionary terms, an ability to identify embedded figures would, clearly, be required to distinguish any partially camouflaged predators (Llewellyn and Hobson, 2015). Moreover, the visual sense is the only modality which readily enables non-obvious associations to be made, incorporated into a whole and readily communicated (Paivio, 1990, 2013). Indeed, as noted earlier, the prime function of mental imagery may be to make predications. Ever since the Aserinsky and Kleitman (1953) and Dement and Kleitman (1957) studies, human visual-like processing of mental imagery in REM dreaming has been of sustained interest.

During REM dreaming, Aserinsky and Kleitman (1953) first drew attention to the eponymous REMs, but their exact function is still unresolved. There has been an extended debate as to whether REMs are functionally similar to saccades in wake and relate to mental imagery in dreaming, for review, see (Miyauchi et al., 2009). However recent work has made some progress on the function of REMs. In goal-oriented dreams with motor behavior, REMs were directed toward the dream events (Leclair-Visonneau et al., 2010). Sprenger et al. (2010) found that when people imagine vivid scenes during dreaming, their REMs are similar to eye movements with open eyes when imagining remembered scenes in wake. Although, research on REMs has tended to dichotomise their function as either scanning (Leclair-Visonneau et al., 2010) or generating visual (dream) imagery, other work indicates that REMs do both (Hong et al., 2009). In the absence of sensory input (as during REM dreaming), imagination is required to generate a mental image (Conway, 2009; Nir and Tononi, 2010). As discussed earlier, predicting the future relies on the imaginative association of elements of past experiences (Szpunar et al., 2007; Addis et al., 2009; Addis and Schacter, 2011) to identify the patterns which enable predictions.

Friston et al. (2012) argue that saccades are directed visual searches to generate sensory evidence for a hypothesis formed on the basis of previous experience. For example, the hypothesis may be that movement in the undergrowth surrounding the waterhole is caused by a “sit and wait” predator. Similarly, REMs may be directed visual-like searches of memories to generate evidence for a prospective code which can be mobilized in the future wake state as a predictive code or perceptual hypothesis. Such a conclusion is congruent with Hong et al. (2009) who argue that REMs both scan and generate dream imagery. It is also compatible with Andrillon et al. (2015) who demonstrate that saccades and REMs are both “reminiscent of visual-mnemonic responses” and both occur at transitions in visual (or visual-like) processing. Such transitions may, in REMs, trigger a new dream image.

As noted by Friston et al. (2012) active sampling reflects the self-organizing nature of the brain. The idea that REMs both scan and generate dream imagery is also a self-organizing principle. Self-organizing systems, like the brain, are characterized by circular causality which is not tautologous (Kelso, 1997; Freeman, 1999; Hardy, 2001; Keller, 2007). Much of contemporary physiology and psychology still holds to the linear cause and effect assumptions of “inputs and outputs, stimuli and responses,” self-organizing poses a fundamental challenge to these suppositions (Kelso, 1997) and speaks to the constructive aspect of perception as exemplified, par excellence, in dreaming.

### REM dream bizarreness and prospective coding

McCarley and Hoffman (1981) reported that 67% of REM dreams exhibit bizarreness, i.e., incongruities, indeterminacy or discontinuities, see below and discussion in Rittenhouse et al. (1994). Bizarreness may be caused by the conflation of memory elements (Cicogna and Bosinelli, 2001). As noted above, prospective coding in REM dreaming may conflate memory elements which have the same meaning for the dreamer. For example, in my illustrative dream below I conflate failing an eye test with a saggy, old, toy, cloth cat because, to me, they both mean visual field restriction. This conflation engenders a bizarre dream image. The term “bizarre” describes a place, person or object which assumes multiple (seemingly inconsistent) elements. However, judgements about bizarreness (or inconsistency) in dreams are made from the wake state. As discussed above, Stickgold et al. (1999) found changes in associative memory systems across wake, NREM and REM where the normal pattern of priming (more response to strong primes) was demonstrated in wake and on awakening from NREM sleep whereas there was more response to non-obvious or “looser” primes on awakening from REM sleep. This research implies that the mind in wake may not easily identify the “looser,” non-obvious associations made in REM dreaming, giving rise to judgements that the associations made in dreams are “bizarre.” Whereas it may be that REM meaning-based associations are more creative because they are non-obvious but, at least potentially, discernible in the wake state. Edwards et al. (2013) found that dream analysis gave rise to “Aha!” insights in relation to both the associative nature of the dream content and its memory source. Indeed, the very use of the term “prospective *coding*” implies opacity but also the possibility of discerning the code.

Rittenhouse et al. (1994) argue that bizarreness in dreams operates under constraints which limit it to incongruities (inconsistent or fused features of people, objects, or places), indeterminacy (where the identity of a person, object or place remains explicitly vague) and discontinuities (sudden changes in these features of people, objects or places). As argued above, the inconsistency may be apparent, arising from the propensity of the mind in wake to be more attuned to obvious, rather than non-obvious, more creative, associations.

Hobson and Friston (2012) suggest that sleep reduces model complexity and prunes redundancy to optimize predictive coding during wake (in formal statistical terms complexity minimization is known as Occam's principle), see also Hobson et al. (2014). During REM dreaming, a reduction in prospective coding complexity may be achieved, in part, through fusion of memory elements with the same meaning for the dreamer, which elicit similar emotions in the dreamer and may require the same motor responses—if used as a predictive code in wake. Similarly, pruning of redundancy during sleep may be achieved through rendering the identity of people, objects, or places vague—where their explicit details are not required to generate an effective image-based prospective code. Indeed, including such non-relevant details would distract from the essential “message” of the code for new situations. Indeed, eliminating redundant aspects of internal models ensures generalization to new circumstances. A good internal model only captures the essentials of what is important for (my) inferences about the world.

Both of these aspects of pruning redundancy (fusion and vagueness) enable abstraction or generalization-as noted by Hobson et al. (2014). Pruning redundancy can be conceptualized as de-contextualization which, as noted by Horton and Malinowski (2015), also enables generalization through stripping away the original context. For example, although a lion and a hyena are different they both belong to the more abstract category of “predators.” They also both inspire fear and require the same motor response of freeze or flight. Fusing a lion and a hyena in a “liena” associative image would generalize a prospective code to more than one predator. Similarly if both a lion and a hyena had been sighted on separate occasions on approach to a waterhole, memories of these associated waterhole events may be rendered vague in a prospective code- excepting for the relevant predator focus.

The scene discontinuities which Rittenhouse et al. (1994) identify as bizarre may, in a similar manner to the inconsistencies, reflect non-obvious associations or themes which link dream scenes. Specifically, a peripheral association in one scene may become dominant in the following scene (Llewellyn, 2013), and the continuity may have an emotional basis (Nielsen, 2013).

In the next section I use a dream to illustrate (a) how pattern identification amongst dream memory elements may enable prospective coding and also, sometimes, fuse associated memory elements (b) how vagueness in dreams may be engendered by pruning redundancy and (c) how apparent scene discontinuities may result from transitions between image-based codes which are connected by an underlying emotional theme.

The illustrative dream was collected in the home and recorded in a dream diary. There are no claims that it is typical in any way. It is self-identified as a REM dream because it conforms more to REM dream characteristics than NREM. Distinctions between REM and NREM dreams are debated and differences may resemble a continuum rather than reflect discrete binary oppositions. Nevertheless, REM dreams have been found to be more story-like, hyperassociative and bizarre, spatially defined, animated, characterized by vivid internal imagery, driven by primary emotions (such as fear, anxiety, and elation) and typified by more threatening interactions than NREM dreams, which are shorter, more literal, and thought-like, perseverative, verbal, and feature more friendly interactions (Lavie et al., 1984; Foulkes et al., 1989; Cavallero et al., 1990; Stickgold et al., 1994; Nielsen, 1999, 2000; Nielsen et al., 2001; Hobson, 2002; Fosse et al., 2004; McNamara et al., 2005; Desseilles et al., 2011).

## Illustrative dream - “Bagpuss”

The analysis of this dream encompassed two related processes: memory sources were identified and patterns in remembered past events were isolated.

The memory sources of dreams are, often, non-obvious (Nielsen and Stenstrom, 2005). Dream reports are also open systems, being always potentially open to additional or new interpretations. In consequence, a hermeneutic approach was used to identify memory sources and patterns for “Bagpuss.” Hermeneutic inquiry is a textual method (where the “text” can be a remembered event) for open systems which is appropriate when associations are not readily apparent and, therefore, have to be recovered through iterative processes of projection and modification (Ricoeur, 1974, 1981).

For a dream report, it is not possible to “falsify” (Popper, 1959) an identified memory source or pattern. Additionally, the hermeneutics of recovery do not seek to identify the “world behind the text” or the hidden intention, desire or fear of the (dream) author but the “world in front of the text”—which are phenomena (associations, patterns in past events) of which the author could be aware but the construction of the text has obscured (Bauman, 2010). When the text is a dream (or dream report), Bauman's ideas imply that the dreamer has not intentionally disguised the memory sources of the dream (cf. Freud) but the hyperassociative and decontextualizing process of dream construction has masked the memories which constitute the dream.

The hermeneutic process of recovery is essentially reconstructive. Reconstruction characterizes memory recall which shares the same neural networks as imagining the future and navigating, see, for example (Bartlett, 1932; Schachtel, 1947; Conway and Pleydell-Pearce, 2000; Hassabis and Maguire, 2007; Schacter and Addis, 2007). As discussed earlier, dream analysis gives rise to “Aha!” experiences both in relation to memory sources and associative patterns in the content (Edwards et al., 2013). “Aha!” moments occurred during the analysis of “Bagpuss” and were experienced as insights into memory sources or patterns but, as discussed here, insights cannot be taken as definitive.

“Bagpuss”

Date: July, 2009

Scene 1

I am waiting with PB in the Eye Hospital. I'm in the main part of the hospital but I am (kind of) aware that I have to go somewhere else. I tell a nurse that I can't see properly, that my vision isn't right. She says that I have to see someone in the other building. I am anxious about getting there and getting lost.

Scene 2

I am in another building and walking toward some carpeted steps. Someone (a man?) is close behind me, is he helping me or threatening me in some way? I am getting anxious again. He is gray-haired. I think he is East European, maybe he is a Pole? Also he may be the ophthalmologist.

Scene 3

I am sitting at a desk. The ophthalmologist is testing my eyes. He produces a saggy, stripy cat- a kind of large, soft toy. But I can't see properly what it is. I have to identify some black signs (like the letters on an eye chart but they may not be letters) that are under the cat's skin. I can't see them properly. So the ophthalmologist pushes the toy cat closer to show me. I know that I have failed the eye test. I feel very anxious.

### The possible associated source memories for “Bagpuss”

In July 2009 a few evenings previous to the Bagpuss dream I went out to dinner with my partner and D, his brother. D was talking about walking in the countryside with his “pole.” I felt confused and had to ask for clarification because, prior to this conversation I had never heard of a walking stick being called a “pole.” For some reason, the “pole” discussion which ensued caught my imagination.

About 2 weeks prior to this dream I went to the Eye Hospital to have a test for glaucoma. I was very anxious about this test. Glaucoma results in loss of peripheral vision and is common in the elderly. I usually get my eyes tested at the local optometrist. This was the first time I had been back to the Eye Hospital since I was a child. The visit made me feel old. I went to the building that I used to visit as a child but, on approaching the steps, realized that this old building (although still having the sign “Eye Hospital”) was actually closed down. The new Eye Hospital was around the corner but I was uncertain exactly where. Scene 1 reflects this- in the sense that in waking I went to the wrong building. In the dream, however, I am in the original Eye Hospital building and see a nurse. PB did not go with me to the Eye Hospital either as a child or an adult. I worked with PB, however, in the Main Hospital—which adjoins the old Eye Hospital when we were both young women.

**Figure 1:**
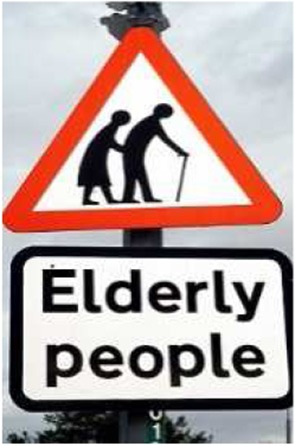
**Sign used in the UK to indicate that elderly people may be crossing the road**.

The steps I approach in Scene 2 could reflect the ones at the entrance to the old Eye Hospital. The positioning of the gray-haired man and me in Scene 2 resembles the sign used in the UK to indicate that elderly people may be crossing the road (an old man leaning on a walking stick with a bent old woman immediately behind him; Figure 1).

In the dream, the gray-haired man is behind me. Indeed in wake I had misremembered this aspect of the sign because on seeing it on the internet I surprised that the woman is behind rather than in front of the man. The East European (Pole) reference may be a memory of East European immigration which (at the time) was a political issue in the UK.

In Scene 3 the action resembles memories of many of the eye tests I have undergone in the sense that there is an ocular specialist, a means of eye testing and me.

### Pattern identification amongst memories may enable prospective coding and, as a result, engender bizarreness

As discussed above, Scene 1 may portray associations amongst my recent appointment at the Eye Hospital with the ones I had as a child where I did enter, wait and then see a nurse. As discussed above, prospective coding must rely on discerning patterns in the world. The pattern, here, may be “not being able to see” accompanied by anxiety. As a child I had to remove my glasses to have my eyes tested whereas as an adult I take out contact lenses but in both cases because I am so short-sighted it engenders anxiety about moving around. Another possible related pattern is “fear of blindness.” As a child, I wore glasses from the age of five. I used to fear that the increasingly strong spectacles I was prescribed indicated I may go blind. As an adult I am being tested for glaucoma which can cause blindness.

In Scene 2 the non-obvious pattern appears to be “pole” or “Pole.” Various associations to “pole” may be distinguished in this scene. Blind people, like the elderly, need sticks or “poles” to walk. The gray-haired man behind me who is helping me to walk (or may be threatening in some way) may reflect the elderly man in the road sign using a walking stick or “pole.” One of my former colleagues, who specialized in geriatrics, very much disliked this sign. She said that it stigmatized older people as bent and incapable and also looked as if one was a pickpocket- in that it portrayed the couple as though one was taking money from the other, this association may explain why, in the dream, I am uncertain if the man is helping or threatening. At the time of the dream, many UK nationals thought Poles were a threat to “their” jobs and were, therefore, taking money from them. This issue was much discussed on the news.

In Scene 3 the non-obvious pattern may be “loss of peripheral vision.” The eye chart is fused with a soft, cat toy creating a bizarre image. “Bagpuss” is a 1970s children's television character: an old, pink striped, saggy cloth cat. Bagpuss is always shown in scenes that fade out into hazy, blankness (see, Bagpuss on youtube, https://www.youtube.com/watch?v=dpwhohWhrEE). Glaucoma involves a loss of peripheral vision. Therefore, in the sense of an image-based code for visual field restriction, Bagpuss is a non-obvious association with glaucoma. To me, Bagpuss and glaucoma both *mean* visual field restriction.

### Vagueness in dreams may be engendered by pruning redundancy

In “Bagpuss” vagueness, or ambiguity, is most apparent in Scene 2. In terms of reducing model complexity and pruning redundancy (Hobson and Friston, 2012; Hobson et al., 2014), prospective coding in sleep may induce fusion (as described above) *and* vagueness because both would reduce the complexity of the visual image, which, in turn, would facilitate rapid deployment in wake. Complexity is reduced through pruning detail from the memories involved- whenever this detail is not required for the associations which are being portrayed in the dream. In this sense, “pruning” is analogous to memory condensation through association, noted by Freud. In Scene 2 there are three ambiguities: the man is behind me and, therefore, not visible and yet I next note his gray hair, implying I have seen him; his intentions are unclear- is he assisting me or menacing in some way: and his identity is ambiguous, is he a “Pole” or the ophthalmologist or is he a Polish ophthalmologist. And yet, from the perspective of associational meaning, the image is, arguably, unambiguous. It portrays my associations to “poles/Poles.”

### Scene discontinuities may mark transitions to a new image-based prospective code but have an underlying emotional theme

Bagpuss has a story-like progression. I am looking for a place in the Eye Hospital in Scene 1, in Scene 2 I may be approaching that place and in Scene 3 my eyes are being tested. Nevertheless, there is discontinuity in the sense of the abrupt transitions between scenes. As discussed earlier, Andrillon et al. (2015) found that REMs, like saccades in wake, signify transitions in image-based or visual processing. Therefore, REMs may generate new dream scenes which may constitute linked, prospective image-based codes. All three scenes may be occurring at the Eye Hospital which, for me, may be an emotionally-charged “landmark” place (see below). The emotive theme of “vision loss or failure” also links the three scenes. This is less apparent in Scene 2 than the other two scenes, albeit there is a reference to an ophthalmologist. However, Scene 2 is concerned with “poles” which are needed by the blind or the elderly to move about. And the “Pole” appears to be linked to “poles” through the associated pickpocket threat (as perceived by my former colleague) in the “elderly people crossing” sign and the perception held by some UK Nationals that “Poles” are taking “their” jobs and, therefore, “their” money.

### Summary of prospective coding during REM sleep and dreaming

Through discerning patterns inherent in prior experiences (and knowledge), REM dreaming may portray the associations between the memory elements which make up the pattern. These associations may constitute new memory content. During REM sleep, Landmann et al. (2015) report that memory “reorganization” results in novel (i.e., new) memory content which was not encoded directly during wake. This new associational memory content may enable predictions though constituting image-based prospective codes which can mobilized as predictive codes during wake.

A self-organizing brain may both engender and identify meaning through making associations. As argued earlier, meaning is often idiosyncratic to the individual, being derived from prior experiences (and knowledge) and the individual's needs, desires, and goals—all of which may be opaque- even to those intimate with them. For this reason, the dreamer should have privileged insight into meaningful dream associations, which is not equivalent to asserting that the dreamer is necessarily correct. Hill et al. (1993) found interpreting one's own dream engendered more insight than interpreting that of another person, see also Edwards et al. (2013) and Edwards et al. (2015) for dream insight studies.

In psychology the term “elaborative encoding” designates the process of discerning meaning in past events and the binding together of meaningfully associated elements of past experience to enable inferences to be drawn (Craik and Tulving, 1975; Tulving, 1983; Craik, 2002). Llewellyn (2013) argues that REM dreaming is elaborative encoding during sleep. This may be another way of contending that REM dreaming constitutes prospective coding because elaborative encoding enables inferences which, in turn, generate predictions. Indeed, to “encode” may imply generating the meaningful associations which constitute a “code.” Theta oscillations occur during associative mnemonic processes (Miller, 1989, 1991), concomitantly, theta is essential to encoding (Buzsáki, 2002; Vertes, 2005). A theta-gamma code underlies the associative encoding of multiple items or events and their contexts (Lisman, 2005; Lisman and Buzsáki, 2008; Tort et al., 2009; Lisman and Jensen, 2013). Theta-gamma coordination is present during both wake and REM sleep (Montgomery et al., 2008), indicating that encoding may characterize both states, but encoding during REM sleep may be more elaborate (Hutchison and Rathore, 2015) in the sense of the associations created during REM being non-obvious or “looser.”

In evolutionary terms, the creative, non-obvious associations forged in REM dreaming may serve to complement the more obvious, tight associations made in wake (Stickgold et al., 1999). As argued earlier, Wallas (1926) pointed out the evolutionary imperative to recognize “resemblance in difference.” Bagpuss and glaucoma are very different but they are both characterized by visual field restriction. Contemporarily, loss of peripheral vision is a frightening prospect but does not jeopardize my survival. In evolutionary terms, however, such vision loss would have been life-threatening. During REM dreaming when prefrontal regions are deactivated, cognitive control is attenuated which may enable the identification of non-obvious associations (see earlier). Hence, this threat of vision loss may have generated an inclusive search for any “resemblance in difference” associations. Any previous experiences with visual field restriction could constitute part of a meaningful pattern which could possibly assist in coping with the threat.

A related way of explaining the identification of loose associations in REM dreaming is that non-obvious or “bizarre,” personally meaningful, visual associations are mnemonic i.e., they aid memory retention and retrieval (Luria, 1968; Groninger, 1971; Roediger, 1980; McDaniel and Einstein, 1986; Einstein and McDaniel, 1987; Wilding and Valentine, 1997; Wang and Thomas, 2000; Worthen and Hunt, 2011; Llewellyn, 2013).

## Off-line states and prospective coding

As discussed above, during off-line states, people review the past to predict the future (Bar, 2007, 2011; Schütz-Bosbach and Prinz, 2007). Such reviews of the past may assume somewhat dissimilar forms in different off-line states. As discussed above, the off-line state of REM dreaming may engender a prospective code which results from the identification of an associative, non-obvious probabilistic pattern in events, for example, the associative pattern amongst “waterhole” and “abundance of prey” and “presence of a predator.” Off-line states also occur during wake, when, rather than perception of the immediate sensory environment, brain processing turns to cogitation on the past and future. For example, the off-line “default mode” (Binder et al., 1999; Raichle et al., 2001) shows overlap with brain regions activated during the associative processing which may generate predictions (Bar et al., 2007). Brain processing during wake, however, is more attuned to tighter, more logical associations than the looser, non-obvious associations discerned during REM sleep (Stickgold et al., 1999). In turn, this may imply that the patterns identified during wake are more strongly determined by regular co-occurrences or tight *sequences* of events. Therefore, prospective coding during wake may take a different form as compared to prospective coding during REM dreaming- albeit that these different forms may constitute more of a continuum than a dichotomy. Different forms of prospective coding may relate to the ecological context within which prospective coding may have evolved.

Before discussing- in some detail- the possible ecological context for the evolution of different forms of prospective coding, it is worth noting that formal or computational approaches speak to the evolutionary significance of prospective coding. There is a body of work that shows prospective coding is necessary for planning and inference, demonstrated most clearly in the context of Markov decision processes—as a model for planning and choice behavior, see Friston et al. (2015). Crucially, this work indicates that prospective coding enables agents to interrogate affordances (see earlier) to select actions or plans with the greatest epistemic or pragmatic utility. In other words, prospective coding is necessary to both derive knowledge from the environment and obtain pragmatic rewards, which has obvious evolutionary imperatives.

### The ecological context for prospective coding?

We live on a patchwork planet (MacDonald and Johnson, 2015). Natural resources, including food and water, are clumped rather than evenly or randomly dispersed, consuming food from clumped areas requires retained knowledge of their locations (“place memory”), indeed, patchy resource consumption may have driven the evolution of spatial memory (Cunningham and Janson, 2013).

Few animals are nomadic (Powell, 2000). Nearly all occupy confined, fairly stable habitats for a season, a year or even a lifetime, this regularly exploited territory is the ‘home range’ (Burt, 1943), a concept which originates with Darwin (Börger et al., 2008). Within the home range, animals will tend to return to resource-rich locations (i.e., ones with food, water, refuge or mates) whilst trying to avoid places where they have encountered aggression or danger, for review, see Stamps and Krishnan (1999). Such strategies increase fitness through exploiting resources efficiently and avoiding risk (Spencer, 2012).

In consequence, for any animal (or early human), there would have been key landmark places which were used regularly, for example, dependable food sources and refuges from predators (Kaufmann, 1962; Samuel et al., 1985; Benhamou and Riotte-Lambert, 2012). In natural environments, animals still make tours, sometimes daily, between a series of such resource-rich places, where they spend time, followed by a straight return to the home base, indicating memory for what-where place associations and the use of landmark navigation- at least of a rudimentary kind (Janzen, 1971; Gallistel, 1990; Wallace et al., 2006; Noser and Byrne, 2007, 2010).

In such an ecological context, two forms of prospective coding may have evolved, prospective coding which required probabilistic, non-obvious associations amongst prior events at the landmark places and prospective coding of the sequences which constituted the pathways or journeys between the landmark places, see Llewellyn and Hobson (2015). The visual sequences along regularly used pathways or frequently undertaken journeys would, in all likelihood, be more characterized by a tighter associational status than the discontiguous events (e.g., sightings of predators at different times) at the landmark place.

### Prospective coding of landmark places and REM dreaming

Landmark places are characterized by spatially clumped resource availability which attracts many animals and is associated with increased aggressive behavior, for review, see Robb and Grant (1998). For example, animals which drink water must make regular visits to waterholes, rendering them “landscapes of fear” (Willems and Hill, 2009; Hayward and Hayward, 2012) because both prey and predators congregate there (Burger, 2001) and predators often hunt their prey in the vicinity of water (Meer et al., 2012). As mentioned earlier, male lions are “sit and wait” predators, they take advantage of long vegetation around waterholes to ambush prey (Hopcraft et al., 2005; Valeix et al., 2009, 2010). Similarly, snakes select their ambush sites at oases < 5 m from the water (Tsairi and Bouskila, 2004). At waterholes, elephants intimidate, aggressively chase and passively interfere with others present (Valeix et al., 2007). For an animal (or early human) on approach to a waterhole, predicting the cumulative patterned place-reward-danger associations of this landmark would enable appropriate action with respect to risk. If there is only one waterhole available to the animal, it cannot always avoid visiting for fear of predators or aggressive competitors.

On any tour, the animal's approach to a landmark is the most dangerous time (Valeix et al., 2009; Périquet et al., 2010). Congruent with dreaming associating experiences which, in evolutionary time, occurred on approach to a regularly visited landmark place, in a study (not distinguishing between REM and non-REM) 73% of dreams portrayed approach behaviors (Malcolm-Smith et al., 2012). Perogamvros et al. (2013) stated that dreams prioritize significant phenomena which have a high value for survival. The self (the dreamer) features in 95% of REM dreams (Snyder, 1970), McCarley and Hoffman (1981) found that 79% of REM dreams are characterized by movement of the lower-extremities, as would be required for spatial navigation. Typical themes in dream reports may reflect evolutionary “landmark” origins, for example, two studies (not distinguishing between REM and non-REM) reported “being chased or pursued,” “falling,” and “a person now alive as dead” occurring in 92.2, 87.1, and 75.0% of one student sample and 94.6, 76.8, and 69.6% of another (Yu, 2008, 2011). Anxious uncertainty about outcomes is common in dreams (Schwartz, 2012). Dream reports encompass more social interactions than wake reports and REM dreams portray more aggressive interactions than non-REM, the difference was threefold (McNamara et al., 2005). As discussed above, landmark places, such as waterholes, would be “social,” interactive places because they attract mates and competitors but would also harbor threat causing anxious uncertainty because of the predation risk.

Prospective coding of probabilistic events at a landmark place would have survival value. Anticipation would increase the chances of the animal obtaining rewards (e.g., food, water, and mating), whilst avoiding aggression (Valeix et al., 2009). As noted earlier, the imperative to make tours to consume resources at landmark places may have driven the evolution of spatial memory networks (Cunningham and Janson, 2013). Indeed, the basic spatial network characteristic of pathways which meet at omnidirectional landmark junctions may be conserved in human brain networks (Burgess et al., 2002; Llewellyn, 2013; Llewellyn and Hobson, 2015). At an omnidirectional landmark junction, individual pathways are superimposed and, therefore, associated (O'Keefe and Burgess, 1996) in both world and mental space. An omnidirectional junction is comprised of neurons which collectively express its meaning or significance (Buzsáki, 2005). In evolutionary terms, this associative meaning may have had concrete significance for the needs, desires and goals (Clore and Ortony, 2000) of an animal on approach to a landmark junction. REM dreaming may engender a prospective image-based code of any meaningful, associative, probabilistic, experiential pattern in past events at a landmark. On approach to the landmark in wake, in the context of sensory input which could be indicative of threat, this prospective code could be mobilized rapidly and unconsciously as a predictive code to decide whether to continue to approach or to retreat. Prospective coding may also be undertaken for the pathways which link the landmarks, this will, briefly, be considered next.

### Prospective coding of sequences during wake

In contrast to prospective coding during REM dreaming, which may associate and encode “temporally discontiguous events” (Rawlins, 1985; Buzsáki, 1996) to engender an omnidirectional landmark junction, prospective coding during off-line states during wake may encode spatiotemporally ordered sequences which, across evolutionary time, equated to the pathways, journeys or trajectories which linked the landmark places. When traveling along any previously experienced trajectory, sensory data triggers memories of the upcoming places (Jensen and Lisman, 1996) through hippocampal forward “sweeps” (Johnson and Redish, 2007; Lisman and Redish, 2009). This form of prospective coding may rely on tighter, more deterministic, associations than that undertaken in REM dreaming.

### Prospective coding during NREM sleep and dreaming

This paper offers a hypothesis: REM dreaming is one form of prospective coding which evolved from identifying a loose or non-obvious associative pattern in personally significant events at a frequently visited landmark place. Dreaming also occurs during NREM sleep, however. Although, as discussed above, NREM dreams are rather different, being, inter alia, shorter, less hyperassociative, more literal, and thought-like.

It is suggested above that prospective coding during wake may encode associative sequences, evolving from the encoding of the pathways which linked the omnidirectional landmark places. However, the theory of cognitive mapping encompasses landmark locations and *their relationships to one another* (O'Keefe and Nadel, 1978; Maguire et al., 1999; Burgess et al., 2002). Associations between the landmark places would be the routes traveled and their relative directions. Prospective coding during NREM dreams may have evolved from encoding these associations.

There would also have been an imperative to encode associations between what could be found at different landmark places. For example, if there were two waterholes within reach of the animal these would be associated because water was afforded at both. Computer simulation produces cognitive maps encompassing both object-scene and scene-scene associations (Sato and Yamaguchi, 2005). Object-scene-scene associations (such as water at two different waterholes) may be encoded during REM dreams but directional scene-scene associations may be encoded during NREM.

### Testing the hypothesis

The overall hypothesis is testable through observing the consequences of brain lesions which obliterate dreaming- if, indeed, such elimination occurs. For example, Solms (1997) undertook a study of patients who reported loss of dreaming after either bilateral lesions of the ventromesial region or lesions of the parieto-temporo-occipital junction. He observed that these patients became “aspontaneous, inert and apathetic…[and] showed a massive depletion in…libido (appetitive interest)” (Solms and Turnbull, 2002). Such depletion in drive and energy would be expected from a loss in the ability to derive those patterns in past events which enable the anticipation of the future. In turn, loss of the ability to anticipate, indeed, to enact the future (Varela et al., 1991; Di Paolo, 2009) would engender loss of meaning and abolish willed action. A critique of such studies, however, is that patients with such lesions may have lost the ability to remember dreams rather ceased dreaming. Replication is required to address this and extend Solm's findings on the loss of appetitive interest.

## Concluding comments

Prime facie, “dream to predict” sounds implausible, not least because the bizarre events in dreams never materialize in the wake state. But if prediction involves prospective codes which fuse phenomena with the same meaning, given a particular context, and render other phenomena vague, if they are not essential to that context, one can entertain a role for REM dreaming in prospective coding. Dream bizarreness may be an aspect of the prospective coding process rather than a concrete signal of things to come. A prospective code, generated in REM dreaming, may identify a personally salient, non-obvious probabilistic pattern in past events and portray that pattern in an unconscious, sensorimotor image which, if mobilized as a predictive code in wake, supports cognition in wake through rapidly co-ordinating sensory input with appropriate action. The form taken by any prospective code may also relate to the ecological context for the predictive brain, where REM dreaming evolved to engender an image-based code which hyperassociates temporally discontiguous events at what was, in evolutionary terms, a frequently visited landmark place.

Dreams are odd but as Dobzhansky (1973) remarked “Nothing in biology makes sense except in the light of evolution.”

### Conflict of interest statement

The author declares that the research was conducted in the absence of any commercial or financial relationships that could be construed as a potential conflict of interest.
